# An investigation of polymorphisms in the 17q11.2-12 CC chemokine gene cluster for association with multiple sclerosis in Australians

**DOI:** 10.1186/1471-2350-7-64

**Published:** 2006-07-26

**Authors:** Matthew J Bugeja, David Booth, Bruce Bennetts, Robert Heard, Justin Rubio, Graeme Stewart

**Affiliations:** 1 The Institute for Immunology and Allergy Research, Westmead Millennium Institute, Westmead Campus, University of Sydney, Westmead, NSW 2145, Australia; 2 Department of Molecular Genetics, The Children's Hospital at Westmead, University of Sydney, Westmead, NSW 2145, Australia; 3 Discipline of Paediatrics and Child Health, University of Sydney, NSW 2006, Australia; 4 The Howard Florey Institute and the Southern MS Genetics Consortium, University of Melbourne, Parkville, Victoria 3052, Australia

## Abstract

**Background:**

Multiple sclerosis (MS) is a disorder of the central nervous system (CNS) characterised by inflammation and neuronal degeneration. It is believed to result from the complex interaction of a number of genes, each with modest effect. Chemokines are vital to the migration of cells to sites of inflammation, including the CNS, and many are implicated in MS pathogenesis. Most of the CC chemokine genes are encoded in a cluster on chromosome 17q11.2-12, which has been identified in a number of genome wide screens as being potentially associated with MS.

**Methods:**

We conducted a two-stage analysis to investigate the chemokine gene cluster for association with MS. After sequencing the chemokine genes in several DNA pools to identify common polymorphisms, 12 candidate single-nucleotide polymorphisms (SNPs) were genotyped in a cohort of Australian MS trio families.

**Results:**

Marginally significant (uncorrected) transmission distortion was identified for four of the SNPs after stratification for several factors. We also identified marginally significant (uncorrected) transmission distortion for haplotypes encompassing the *CCL2 *and *CCL11 *genes, using two independent cohorts, which was consistent with recent reports from another group.

**Conclusion:**

Our results implicate several chemokines as possibly being associated with MS susceptibility, and given that chemokines and their receptors are suitable targets for therapeutic agents, further investigation is warranted in this region.

## Background

Multiple sclerosis (MS) is the most common chronic neurological disease in young adults. It is characterised by inflammation of the central nervous system (CNS), believed to be the result of an autoimmune reaction resulting in demyelination and destruction of neural supporting cells [1]. Epidemiological studies suggest a multifactorial aetiology for MS, implicating a complex interplay between environmental and genetic factors [2]. In the past ten years, a large number of genome wide screens have been conducted, including the recent GAMES collaboration (Genetic Analysis of Multiple sclerosis in EuropeanS) [3]. Multiple regions of potential linkage and association with MS have been identified, suggesting that genetic predisposition to MS might result from the modest contribution of many genetic factors, which, if identified, may present important new therapeutic targets [4].

The inflammatory response that is characteristic of MS requires the targetted migration of leukocytes into the CNS, which is under the control of chemokines. Over 40 members of the human chemokine family have been identified, which act upon a variety of leukocytes via interactions with almost 20 seven-transmembrane domain chemokine receptors [5]. Chemokines are small molecules of approximately 8–10 kDa in size, and are primarily classified on the basis of the relative positioning of two conserved cysteines. In the CC chemokines, the two cysteines are adjacent, and in the CXC chemokines, a single amino acid residue separates the cysteines. Two smaller subgroups have also been identified; the CX_3_C chemokines, and the C chemokines.

Functionally, CC chemokines chemoattract a wide range of cells, including lymphocytes, dendritic cells, monocytes and some granulocytes [6,7], whilst CXC chemokines are chemoattractant for neutrophils and lymphocytes [6,7]. There is increasing evidence for chemokines possessing abilities beyond that of migration, including T-helper cell subset differentiation [8], T cell costimulation [9,10], and macrophage and natural killer cell maturation [11,12].

Substantial evidence supports the involvement of CC chemokines in the pathogenesis of MS. In the mouse model of MS, experimental autoimmune encephalomyelitis (EAE), knockout of *CCL2 *leads to resistance to disease induction [13], whilst CCL3, CCL4 and CCL5 have all been implicated in EAE development [14,15]. However, CCL3-knockout mice were found to be fully susceptible to myelin oligodendrocyte glycoprotein (MOG)-induced EAE [16]. In MS lesions, expression of CC chemokines, including CCL2, CCL3, CCL4, CCL5, CCL7 and CCL8, and their receptors, have been identified on a wide variety of cells, such as astrocytes, microglia and perivascular T cells [17-22]. In addition, altered levels of CC chemokines and receptors have been identified in the serum and cerebrospinal fluid (CSF) of MS patients; some are elevated (including CCL5), whilst CCL2 is decreased in the CSF [22-26], possibly due to removal by CCR2-positive migrating cells as they cross the blood-brain barrier [27].

**Figure 1:**
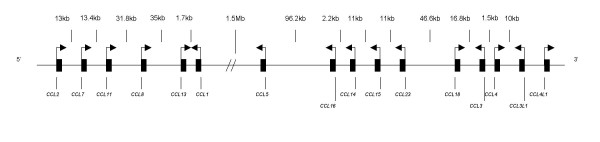
Schematic representation of the 17q11.2-12 CC chemokine gene cluster (not to scale).

Fourteen of the 28 CC chemokine genes are clustered on chromosome 17q11.2-12 (Figure 1) [28]. This cluster spans slightly less than 2 Mb, and is split into two sub-clusters separated by a gap of 1.5 Mb. The 17q11.2-12 region has been implicated in genome-wide screens for linkage and association with MS [29-34], and in a meta-analysis of three genome screens, the most significant nonparametric linkage score was obtained for this region [35]. The 17q region is also syntenic to an EAE quantitative trait locus on chromosome 10, which includes a chemokine gene cluster [36], and non-synonymous polymorphisms in murine *CCL1*, *CCL2 *and *CCL12 *were identified as candidates for the *eae7 *quantitative trait locus [37].

Candidate gene studies of CC chemokines in MS have not been extensive. Rather, the majority of studies have focussed on other diseases. Polymorphisms from several CC chemokine genes have been variably associated with diseases such as tuberculosis [38], HIV [39], arthritis [40], and asthma and atopy [41,42]. The *CCR5*Δ32 mutation has been widely studied in MS. Whilst it was found not to be a general susceptibility factor for MS [43], it may have effects on age of onset or disease progression [44,45]. More recently, several moderate single-loci and haplotypic associations with MS were identified for single nucleotide polymorphisms (SNPs) from the CC chemokine gene cluster [46].

It was the aim of our study to conduct candidate gene analyses for the CC chemokines in order to identify polymorphisms and/or haplotypes associated with MS. We undertook a two-stage analysis. The first stage involved the scanning of the genes for published (online) and novel polymorphisms in several DNA pools by DNA sequencing. From these, 12 candidate SNPs were then individually genotyped in a cohort of MS trio families in the second stage. We also sought confirmation of our findings in an independent cohort of Australian MS families. Marginally significant (uncorrected) transmission distortion was identified for four of the SNPs, as well as for haplotypes encompassing the *CCL2 *and *CCL11 *genes.

## Methods

### Subjects

For the main study, all sporadic MS cases and parents (including 373 MS trio families) were recruited by our facility at the Institute for Immunology and Allergy Research, Westmead Hospital (Australia). The familial DNA pool was derived from MS probands from multicase families obtained from the National Register of Multiple Sclerosis Families (Rex Simmons, Canberra Hospital, Australia). Control individuals were composed of local staff members and spouses of sporadic MS cases. MS patients and controls had the same ethnic composition, and were of similar average age (patients = 50yo; controls = 52.8yo). 90% of MS patients were of northern European origin, while the remainder were of southern European origin. The ratio of female to male patients was 4:1; the control ratio was 1.5:1. Approximately 60% of MS patients were HLA-DRB1*1501 positive; 73% had relapsing-remitting (RR)-MS, 20% secondary-progressive (SP)-MS, 5% primary-progressive (PP)-MS, and 2% progressive relapsing (PR)-MS. An additional cohort of 208 Australian MS trio families was obtained from the Southern MS Genetics Consortium for independent validation of our initial results. All cases were verified as having MS as defined by the Poser criteria [47], and provided written informed consent.

### Pooled DNA sequencing

DNA was extracted from whole blood using a rapid salting out method [48]. Methods for the construction of the DNA pools has been described elsewhere [49]. Four DNA pools were constructed: 217 HLA-DRB1*1501 positive sporadic MS patients; 155 HLA-DRB1*1501 negative sporadic MS patients; 169 MS probands from multicase families; 185 unrelated, unaffected controls. HLA-DRB1*1501 status was genotyped using a SYBR Green assay (Applied Biosystems (ABI)), described elsewhere [50].

DNA sequencing primers were designed to encompass the exons of all genes analysed, as well as segments of the putative promoter region, and often part of the 3' untranslated region (UTR) (see additional file 1: 17qSuppTable1). Where possible, primers were also designed to maximise coverage of published SNPs, which were identified from online SNP databases.

DNA sequencing of the chemokine genes was performed on each of the DNA pools and a single individual, included for SNP allele frequency correction purposes and for potential identification of rare SNPs. PCR products for each gene segment were amplified using the ABI 2× PCR Master Mix, with 50 ng of genomic DNA and appropriate amplification primer pairs (Sigma Genosys) to a final concentration of 5 ng/μL. PCR reactions followed a standard PCR program: 1 cycle of 95°C for 10 minutes; 35 cycles of 94°C for 30 seconds, 62°C for 30 seconds, and 72°C for 1 minute and 10 seconds; a final elongation step of 72°C for 10 minutes. PCR products were purified from the PCR cocktail by use of either the QIAQuick^® ^PCR purification kit (QIAGEN), or ExoSAP-IT (USB Corporation). Sequencing reactions were performed using the ABI Big Dye^® ^Terminator v3.1 Cycle Sequencing Kit. Final samples were sequenced using an ABI 3100 sequence analyser. Sequences were viewed using the ABI PRISM™ EditView software.

### Genotyping in Trio Families

#### SNaPshot

An initial set of seven CC chemokine gene SNPs were selected for individual genotyping in a cohort of 204 MS trio families (Table 1): *CCL2 *-2581, *CCL2 *-2138, *CCL11 *67, *CCL5 *-471, *CCL15 *-1284, *CCL15 *136+88 and *CCL23 *-289. These SNPs were genotyped using the SNaPshot assay, which allows the genotyping of multiple SNPs simultaneously in a single-tube multiplexed reaction, using primer-extension methodology [51] (see additional file 3: 17qSuppTable3 for SNaPshot primer details for each of the seven SNPs).

**Table 1:** Summary of SNPs identified by sequencing of CC chemokine genes in DNA pools that were selected for individual genotyping. Other detected SNPs are described in Supplementary Table 2.

		**Estimated minor allele frequency**	**Estimated relative risk** **(for minor allele frequency >0.15)**		
**Locus**	**rsID**	**Control pool**	HLA-DRB1*1501 **positive pool**	HLA-DRB1*1501 **negative pool**	**Familial pool**

**CCL2**					
**-2581A>G**	**1024611**	**0.5**	**0.9**	**0.8**	**0.7**
**-2138A>T**	**1024610**	**0.3**	**1.2**	**1.5**	**1.5**
**CCL11**					
**-488C>A**	**17735961**	**0.2**	**1.2**	**1.2**	**1.3**
**67G>A (A>T)**^+^	**3744508**	**0.1**	**-**	**-**	**-**
**CCL8**					
**-572C>T**	**3138035**	**0.3**	**1.1**	**1.0**	**1.2**
† 205A>C (K>Q)^+^	3138038	0.2	0.8	0.8	0.7
**CCL5 (-)**					
**-471C>T**	**2107538**	**0.2**	**1.2**	**1.3**	**1.1**
**CCL16 (-)**					
**-595C>A**	**854680**	**0.1**	**-**	**-**	**-**
**CCL14 (-)**					
**-649T>A**	**854682**	0.2*	1.1*	1.0*	1.4*
**CCL15 (-)**					
**-1284A>C**	**854628**	**<0.1**	**-**	**Undetectable**	**-**
**136+88C>T**	**Novel**	**0.3**	**0.8**	**0.6**	**0.5**
**CCL23 (-)**					
**-289A>C**	**854655**	0.2*	0.7*	0.5*	1.0*
**316T>C (M>V)+**	**1003645**	0.2*	1.0*	0.7*	1.0*

All SNPs classified relative to translation start site. Relative risks determined relative to control pool. SNPs in bold were individually genotyped in second stage

(-) Gene encoded in anti-sense direction; SNPs reclassified to account for this

^+ ^Coding change in brackets

^†^The CCL8 205 SNP was originally selected for genotyping by SNPLEX™, but failed the SNPLEX™ algorithm, and was replaced with the CCL8 -572 SNP

* Heterozygous individual correction applied

rsID: SNP identification number

SNaPshot genotyping was performed on the pooled PCR products of each individual, amplified using either the ABI 2× PCR master mix, or the Fermentas 2× PCR master mix (Progen). SNaPshot reactions were optimised in individuals of known genotype. Between 100–200 ng of DNA was used from each individual with the appropriate amplification primer pair (Sigma Genosys) to a final concentration of 5 ng/μL. PCR products were amplified using the standard conditions, as described above. The successful amplification of PCR products was verified on 1% agarose gels. The PCR products for each individual were then pooled, and an aliquot purified using ExoSAP-IT (USB Corporation). To this aliquot was then added 2.5 μL of SNaPshot Multiplex reagent (ABI), and pooled SNaPshot primers as defined in Supplementary Table 3, and the SNaPshot procedure was followed as per the manufacturer's instructions (ABI). Samples were then scanned using an ABI 3100. SNaPshot results were viewed using the GeneScan™ software (ABI) and genotypes determined using the GeneMapper™ software package (ABI).

#### SNPlex™

An additional five CC chemokine gene SNPs were selected for genotyping in the full cohort of 373 MS trio families (Table 1): *CCL11 *-488, *CCL8 *205, *CCL16 *-595, *CCL14 *-649 and *CCL23 *316. These SNPs were genotyped using the SNPlex™ genotyping system (ABI), for which SUPAMAC (University of Sydney, Australia) were contracted. This system allows high-throughput genotyping of up to 48 SNPs simultaneously in a single tube using an oligonucleotide ligation assay. Initially, candidate SNP details were submitted, after which suitability for the assay was determined by running the SNPs through an algorithm. Oligonucleotides were then designed for each SNP, and applied to the DNA samples (500 ng genomic DNA). Samples were scanned using an ABI 3730. Genotyping results were determined using the GeneMapper™ software package (ABI), utilising an allelic discrimination cluster analysis to allocate genotype calls with maximum stringency.

#### Additional genotyping of validation cohort

Genotyping of the additional cohort of MS trio families was contracted to the Australian Genome Research Facility (AGRF; Brisbane, Australia). Only the *CCL2 *-2138A>T and *CCL11 *-488C>A SNPs were genotyped using the Sequenom Autoflex Mass Spectrometer. After submission of SNP details, PCR oligonucleotides were designed and applied to the DNA samples.

#### Analyses & statistics

Sequencing results from DNA pools and individuals were compared and scanned for published SNPs and novel polymorphisms. All SNPs were classified according to the nomenclature recommended by den Dunnen and Antonarakis [52], and are relative to the translation start site. For genes transcribed in the anti-sense direction (indicated in Table 1 with (-)), the complementary nucleotide for each allele was used, and is used throughout this manuscript. Minor allele frequencies (MAF) of SNPs were estimated by comparing the relative peak heights of the alleles. Where the individual was heterozygous for a particular SNP, the MAF in the DNA pools could be corrected for SNP-specific variation in peak-height intensity. The significance of differences between the MS patient pools and the control pool was measured by estimated relative risk (ERR) calculations.

Each SNP was analysed for transmission distortion within the trios using the transmission disequilibrium test (TDT) [53]. Analysis was performed using the GENEHUNTER program [54].

The HelixTree genetics analysis software (Golden Helix Inc, Bozeman, USA) was used to calculate linkage disequilibrium (LD) for the 12 SNPs analysed and to determine haplotypes in parents only. By using the Expectation/Maximisation (EM) algorithm, the software was able to calculate probabilities of each haplotype occurring, based on multi-locus genotypes. It calculated D' and r^2 ^values, which are accepted measures of LD, and performed a χ^2 ^comparison for each pair of SNPs. HaploBlockFinder [55] was used to establish the haplotype block structure across the CC chemokine gene cluster.

TRANSMIT [56] was then used to analyse for haplotype transmission disequilibrium, estimating χ^2 ^values for individual haplotypes, as well as global χ^2 ^values for all haplotypes analysed. Mendelian transmission was checked using MERLIN [57].

## Results

### Identification and analysis of SNPs in CC chemokine genes by DNA pool sequencing

The CC chemokine genes were scanned using DNA pool sequencing in order to identify common polymorphisms in the region, including the potential identification of novel SNPs, and to establish whether associations might exist for these SNPs. In all, 50 SNPs were identified across the CC chemokine gene cluster (a list of all variants identified is available in the additional file 2: 17qSuppTable2), 48 of which were known; novel SNPs were detected in *CCL8 *and *CCL15*. Five common SNPs within exons were confirmed, four of which cause codon changes. Twenty-nine SNPs had MAF >0.15 in the control pool, designated as 'common' here, and for which we had statistical power to detect associations for ERR >1.5.

The greater than 90% DNA sequence homology between *CCL3 *and *CCL3L1*, and between *CCL4 *and *CCL4L1*, made it impractical to design specific primers for each of these genes. Thus, these four genes were not sequenced.

### Population genetics of individually genotyped markers

Based on their likely functional significance (codon changing, putative promoter region, reported functional significance) and ERR from the pooled DNA sequencing, 12 SNPs were chosen for genotyping in the MS cohort, either by SNaPshot or SNPlex™ (Table 1). The *CCL8 *205 SNP was originally chosen for analysis by SNPlex™, but failed to pass the SNPlex™ algorithm. Thus, it was replaced with the *CCL8 *-572 SNP, which is in LD with the original SNP (International HapMap Project [58]).

Genotype information retrieval for the seven SNPs genotyped using SNaPshot was 100%. Genotype information available for single-locus analysis for the five SNPs genotyped by SNPlex™ ranged between 84–87%; losses due to failed genotyping, and the exclusion of genotypes called with low stringency. The SNPlex™ genotyping, which is based on a highly multiplex PCR, was quite sensitive to DNA quality. Unambiguous genotypes could be determined using the SNPlex™ calling algorithm, which we have used at the highest stringency. The genotype frequencies for all SNPs conformed to Hardy-Weinberg equilibrium estimates, except for the *CCL11 *-488 SNP in mothers. The Mendelian error rate for the seven SNPs was ≤ 2%. Table 2 lists the MAF of the 12 markers as determined by individual genotyping.

**Table 2:** Common haplotype and minor allele frequencies for SNPs within haplotype blocks in unaffected parents (n = 350–650 individuals), determined by individual genotyping.

**(a) **5' block				
*CCL2 *-2581A>G	*CCL2 *-2138A>T	*CCL11 *-488C>A	*CCL11 *67G>A	Haplotype Frequency
Haplotypes				
A	A	C	G	0.33
G	A	C	G	0.29
A	A	A	A	0.19
A	T	C	G	0.18

Minor allele frequency				
G	T	A	A	
0.29	0.19	0.19	0.20	
	
**(b) **3' block				
*CCL14 *-649T>A	*CCL15 *-1284A>C	*CCL23 *316T>C	*CCL23 *-289A>C	Haplotype Frequency

Haplotypes				
T	A	T	A	0.76
A	A	C	C	0.12
A	C	C	C	0.07

Minor allele frequency				
A	C	C	C	
0.23	0.08	0.20	0.20	
	
**(c) **Minor allele frequencies of remaining SNPs				
SNP	Minor allele frequency			
			
*CCL8 *-572C>T	T 0.37			
*CCL5 *-471C>T	T 0.19			
*CCL16 *-595C>A	A 0.22			
*CCL15 *136+88C>T	T 0.05			

### Linkage disequilibrium and haplotypes

**Figure 2:**
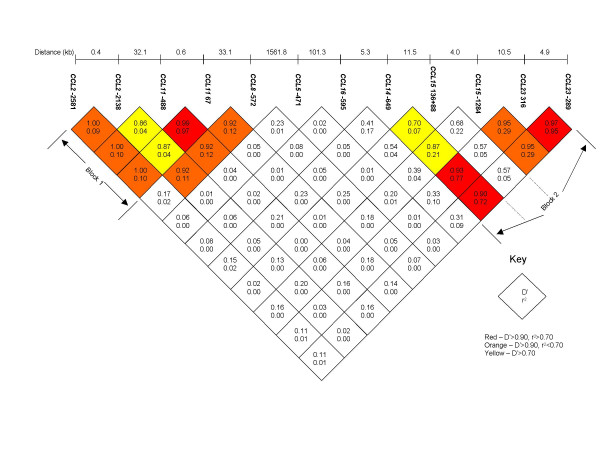
LD plot of CC chemokine gene cluster SNPs.

The HelixTree genetics analysis software (Golden Helix Inc, Bozeman, USA) was used to conduct a pair-wise analysis of LD across the 12 CC chemokine gene markers, using the parents only. This software calculated both D' and r^2 ^measures of LD, and the data are represented in Figure 2. These results were confirmed using the Haploview software package [59]. Results suggest a clear separation of the SNPs into two haplotype blocks of moderate to strong LD, confirmed by HaploBlockFinder [55] (represented in Figure 2). The first block extended from the *CCL2 *-2581 SNP to the *CCL11 *67 SNP; a distance of 33.1 kb. The second block extended from the *CCL14 *-649 SNP to the *CCL23 *-289 SNP, but did not include the *CCL15 *136+88 SNP, which interestingly was not in LD with any of its neighbouring SNPs. This block extended across 30.9 kb.

The HelixTree software was then used to construct haplotypes only across the two clear haplotype blocks, in parents only (350–650 individuals; actual numbers varied for each SNP); and to estimate haplotype frequencies. Table 2 lists the common haplotypes (frequency >0.05) across the two haplotype blocks and their frequencies within the unaffected parents. Four common haplotypes were identified in the 5' haplotype block, with frequencies between 0.18 and 0.33. In the 3' haplotype block, three common haplotypes were identified, but the TATA haplotype was the most common by a substantial margin (frequency = 0.76).

### Analysis for transmission distortion across single markers and haplotypes

#### Single-locus transmission disequilibrium

Analyses for transmission distortion were undertaken for the 12 chemokine gene markers in all of the trios, as well as subgroups stratified based on HLA-DRB1*1501 status, disease course and gender. Results are listed in Table 3.

**Table 3:** Single locus TDT analysis of individually genotyped CC chemokine gene markers.

Locus	All families		HLA-DRB1*1501 positive		HLA-DRB1*1501 negative		RR-MS		SP-MS		Male		Female	
	T:N	P	T:N	P	T:N	P	T:N	P	T:N	P	T:N	P	T:N	P

*CCL2 -2581A>G	79:89	0.4	48:57	0.4	31:32	0.9	51:68	0.1	21:16	0.4	16:13	0.60	63:76	0.3
*CCL2 -2138A>T	56:75	0.1	35:41	0.5	21:34	0.08	41:56	0.1	12:15	0.6	13:13	1	43:62	0.06
*CCL11 *-488C>A	88:96	0.6	45:59	0.2	43:37	0.5	68:69	0.9	15:17	0.7	20:22	0.8	68:74	0.6
*CCL11 67G>A	71:65	0.6	39:37	0.8	32:28	0.6	54:46	0.4	14:13	0.8	15:15	1	56:50	0.6
*CCL8 *-572C>T	143:127	0.3	84:78	0.6	59:49	0.3	107:96	0.4	23:22	0.9	28:32	0.6	115:95	0.2
*CCL5 -471C>T	64:70	0.6	45:35	0.3	**19:35**	**0.03**	47:50	0.8	14:13	0.9	14:18	0.5	50:52	0.8
*CCL16 *-595C>A	103:91	0.4	58:53	0.6	45:38	0.4	76:66	0.4	14:19	0.4	19:19	1	84:72	0.3
*CCL14 *-649T>A	100:88	0.4	64:49	0.2	36:39	0.7	**81:56**	**0.03**	**9:22**	**0.02**	17:21	0.5	83:67	0.2
*CCL15 136+88C>T	18:10	0.1	11:6	0.2	7:4	0.4	15:6	0.05	2:3	0.7	2:3	0.7	16:7	0.06
*CCL15 -1284A>C	29:27	0.8	21:20	0.9	8:7	0.8	25:17	0.2	2:5	0.3	3:6	0.3	26:21	0.5
*CCL23 *316T>C	85:90	0.7	54:57	0.8	31:33	0.8	70:58	0.3	**8:21**	**0.02**	13:22	0.1	72:68	0.7
*CCL23 -289A>C	62:71	0.4	42:42	1	20:29	0.2	51:46	0.6	9:17	0.1	**8:19**	**0.03**	54:52	0.9

T:N = Transmitted:Non-transmitted for major allele

*Markers genotyped using SNaPshot in 204 trio families; HLA-DRB1*1501 positive: 123 trio families; HLA-DRB1*1501 negative: 81 trio families; RR-MS: 147 trio families; SP-MS: 42 trio families; Male: 38 trio families; Female: 166 trio families Remaining markers genotyped using SNPlex™; All families: 269–296 trio families; HLA-DRB1*1501 positive: 156–176 trio families; HLA-DRB1*1501 negative: 113–120 trio families; RR-MS: 193–218 trio families; SP-MS: 52–56 trio families; Male: 53–60 trio families; Female: 216–236 trio families. Significant P values in bold. P values are not corrected for multiple comparisons.

No significant transmission distortion was found for any of the SNPs when analysed in all individuals. Upon stratification, a trend towards excess transmission of the *CCL5 *-471T allele was found in the HLA-DRB1*1501 negative group (P(uncorrected) = 0.03). Interestingly, DNA sequencing had also suggested an increased ERR for the T allele in the HLA-DRB1*1501 negative pool. For the *CCL14 *-649T>A SNP, transmission distortion of the T allele was found in patients with RR-MS (P(uncorrected) = 0.03). In contrast, transmission distortion of the A allele was found in SP-MS patients (P(uncorrected) = 0.02). Transmission distortion in SP-MS patients was also found for the *CCL23 *316C allele (P(uncorrected) = 0.02). For the *CCL23 *-289A>C SNP, transmission distortion of the C allele was found in male MS patients (P(uncorrected) = 0.03). This distortion was accentuated in male patients with SP-MS (P(uncorrected) = 0.007), however this result was inconclusive given the small number of individuals in this group (n = 10 informative transmissions). None of these results would survive a conservative Bonferroni correction for multiple comparisons.

#### Haplotype TDT

Whilst single-locus analyses did not suggest that any of the SNPs were significantly associated with MS susceptibility, it is conceivable that they may define MS-associated haplotypes. Using the TRANSMIT [56] software, haplotype transmission was evaluated for common haplotypes (>5% frequency) of decreasing size from either block, as well as pairwise analyses across all twelve SNPs. Analyses were conducted only where genotyping information was available for all individuals across all 12 SNPs (n = 162 families), with additional trio families removed after identification of Mendelian inconsistencies.

Table 4 lists common haplotypes for which transmission distortion was identified. All of these findings were identified within the 5' haplotype block (*CCL2 *-2581.*CCL2 *-2138.*CCL11 *-488.*CCL11 *67). Marginally significant transmission distortion was found for two four-marker haplotypes, and several three-marker haplotypes (P ≤ 0.05). Marginally significant transmission distortion was also identified for pairwise analyses for *CCL2 *-2581.*CCL2 *-2138 (P(uncorrected) = 0.05) and *CCL2 *-2138.*CCL11 *-488 haplotypes (P(uncorrected) = 0.04). No significant transmission distortion was found for haplotypes across the 3' haplotype block.

**Table 4:** Haplotype TDT of CC chemokine gene cluster SNPs.

Haplotype	Frequency	Transmitted	Non-transmitted	χ^2^(1df)	P (uncorrected)
(a) Local cohort					

5' haplotype block					
4-marker haplotypes					
*CCL2 *-2581.*CCL2 *-2138.*CCL11 *-488.*CCL11 *67 (n = 162 trio families)					
A.A.C.G	0.33	57.0	85.0	5.50	0.02
A.T.C.G	0.18	61.0	40.0	4.36	0.04
3-marker haplotypes					
*CCL2 *-2581.*CCL2 *-2138.*CCL11 *-488 (n = 162 trio families)					
A.A.C	0.33	56.0	86.0	6.33	0.01
A.T.C	0.18	61.0	40.0	4.36	0.04
*CCL2 *-2138.*CCL11 *-488.*CCL11 *67 (n = 162 trio families)					
A.C.G	0.63	70.1	92.0	2.96	0.09
T.C.G	0.18	60.9	40.0	4.34	0.04
2-marker haplotypes					
*CCL2 *-2581.*CCL2 *-2138 (n = 204 trio families)					
A.A	0.52	89.1	117.1	3.80	0.05
*CCL2 *-2138.*CCL11 *-488 (n = 162 trio families)					
A.C	0.62	69.2	93.1	3.50	0.06
T.C	0.18	60.8	39.9	4.32	0.04

(b) Independent cohort (n = 169 trio families)*CCL2 *-2138.*CCL11 *-488					

A.C	0.63	75.1	86.0	0.74	0.4
T.C	0.20	59.9	52.0	0.56	0.5

(c) Combined dataset (n = 331 trio families) *CCL2 *-2138.*CCL11 *-488					

A.C	0.62	144.4	179.1	3.73	0.05
T.C	0.19	120.6	91.9	3.89	0.05

#### Verification of haplotype results in an independent cohort

Independent validation of the results discussed above was sought in an independent cohort of 208 Australian MS trio families obtained from the Southern MS Genetics Consortium. It was determined that genotyping the *CCL2 *-2138A>T and *CCL11 *-488C>A SNPs was sufficient for information extraction across the four markers within the 5' haplotype block.

Genotype and minor allele frequencies for both SNPs were equivalent to those obtained for our cohort, and were in Hardy-Weinberg equilibrium. LD properties between the two markers were also in agreement with our results. No evidence for single-locus transmission distortion was identified for either SNP, supporting our original findings. Full haplotype transmission data could be obtained for 169 trios; losses were due to genotyping failure for either SNP. Two-marker haplotype analysis revealed slight transmission distortion for the A.C and T.C haplotypes in the same direction as our original findings, but the distortion was not significant (Table 4(b)). However, combining the total datasets of the two cohorts restored the original trends for transmission distortion for the A.C and T.C haplotypes (P = 0.05) (Table 4(c)).

## Discussion

In this study, we have analysed the members of the CC chemokine gene cluster for association with MS. Variant chemokine expression could diminish or enhance the inflammatory response characteristic of MS. The majority of CC chemokine genes are located in a chromosomal region (17q11.2-12) that has shown suggestive linkage and association with MS in a number of genome wide screens [29-34]. Most recently, in a large genome wide linkage screen, no genome-wide significant results could be identified beyond the MHC [60]. However, suggestive linkage was identified on chromosome 17q23. An important deduction from this work was that linkage studies are under-powered to detect modest associations even in large cohorts.

We utilised a two-stage approach for this study, the first of which was sequencing of four DNA pools, three composed of MS patients and one control DNA pool across the 17q11.2-12 CC chemokine gene cluster. This approach allowed estimation of MAF for all common (MAF >0.15) SNPs across the CC chemokine genes in our Australian Caucasian cohort, and identification of two novel SNPs. This technique has been validated by our group [61]. Twelve SNPs were chosen for further analysis by individual genotyping, based on their likely functional significance (codon changing, location in the putative promoter region), prevalence (MAF >0.1), and/or uneven representation between the MS and control pools.

Overall, a large amount of genetic data has been gathered from pooled DNA sequencing and individual genotyping. The pooled sequencing provided a close estimate of MAF for SNPs across the 17q chemokine gene region, and verified the existence of common SNPs published in online databases. The individual genotyping data from 350–650 unaffected parents allowed the determination of MAF for the 12 SNPs chosen for further study, establishment of LD structure across the region, and calculation of haplotype frequencies in our unaffected Australian cohort. The MAF data facilitate power calculations for additional disease-susceptibility studies, and it and the haplotype data should prove useful in studies of population variation for these immunologically important genes. From individual genotyping, MAF were established for the 12 markers in a large unaffected cohort (up to 600 unaffected parents), whilst LD analysis revealed the haplotype block structure across the cluster, and haplotype frequencies were also established.

Measurement of transmission distortion for each of the 12 SNPs in MS trios provided some evidence for over-transmission of several of the SNPs after stratification, but these trends would not survive a conservative correction for multiple comparisons.

The *CCL5 *-471C>T SNP, which we found might be associated with MS in HLA-DRB1*1501 negative patients, is of potential functional relevance, as it creates a new transcription factor binding site [41], and has shown associations with atopic dermatitis, atopy and asthma [41,42]. CCL5 is an important inflammatory chemokine, with a range of activities upon eosinophils [62], monocytes and activated T cells [63,64], and has been identified repeatedly in the CNS and CSF of MS patients [19,21,22]. Our data also identify CCL14 and CCL23 as warranting further investigation, with marginally significant (uncorrected) trends towards transmission distortion found in RR-MS and SP-MS patients for *CCL14 *-649T>A, and in males for *CCL23 *-289A>C. Little is known for either of these chemokines, beyond basic functionality, and neither has been studied with regard to MS pathogenesis. The *CCL23 *-289A>C SNP was found to affect a potential SP-1 binding site using the TRANSFAC database [65].

Upon analysing for haplotype transmission distortion across the CC chemokine gene cluster, we found suggestive evidence for association of haplotypes encompassing *CCL2 *and *CCL11 *(Table 4), which lie within a haplotype block spanning 33.1 kb. CCL2 has been implicated in MS pathogenesis. It is chemotactic for T cells and monocytes [66,67], is important in the induction of inflammation in EAE [13], has been identified in MS lesions [17-19], and in contrast to EAE studies has been associated with remission of MS [68].

Recently (and subsequent to the completion of our study), a similar study of polymorphisms across the CC chemokine gene cluster was conducted by Vyshkina et al [46]. In this study, 31 SNPs derived from online databases were selected for genotyping in a variety of individuals. The basis of selection of SNPs was not discussed. This is in contrast to our approach, in which an informed decision for SNP selection was based on several criteria, discussed above, with a particular focus on SNPs with potential functional consequences. The SNPs assayed by Vyshkina et al [46] were predominantly non-coding, with no SNPs from putative promoter regions. Fifteen SNPs overlapped with those identified in our pooled DNA sequencing. Two SNPs, both exonic, were individually genotyped in our study; the remainder did not pass the first stage. One exonic SNP was excluded as it was not common, whilst an exonic SNP in *CCL4 *was not analysed as this gene was excluded from our study. Similar to our study, Vyshkina et al [46] found no strong evidence for association with any single locus. In addition, we identified similar LD structure surrounding *CCL2*.*CCL11 *and between *CCL14 *and *CCL23*, and identified haplotypic associations for the *CCL2*.*CCL11 *haplotype block. Whilst the SNPs analysed in this block were different between the two studies, it might be assumed that they are subject to the LD within the block. Thus, whilst we can not specifically say that we have replicated the haplotype associations of Vyshkina et al [46], we would suggest that the determination of haplotype tagging SNPs in this block is justified. It was interesting that our TDT analysis was conducted entirely in sporadic MS cases, whilst that of Vyshkina et al [46] had an emphasis on familial MS, yet both studies found weak association for the *CCL2*.*CCL11 *haplotype block with MS, suggestive of a general MS susceptibility factor within this block. This group has since verified their results in a second-phase study [69]. Thus, in a combined population totalling almost 1000 MS families (331 Australian; 644 North American), the results implicate a haplotype association encompassing the *CCL2*.*CCL11 *markers.

## Conclusion

We have conducted a two-stage analysis of polymorphisms across the CC chemokine gene cluster. We identified novel SNPs in this region, and added further information to the data available on LD structure and haplotypes across the cluster. Interestingly, our best single-locus findings were for promoter SNPs (*CCL5 *-471C>T, *CCL14 *-649T>A, *CCL23 *-289A>C), which might affect the relative expression of these chemokines and lead to downstream effects on leukocyte migration to the CNS, and therefore influence MS pathogenesis. We also confirmed the potential presence of a haplotypic association across *CCL2 *and *CCL11*. Further validation of the association of these SNPs in independent cohorts, and confirmation of their functional significance would support therapeutic targetting of these chemokines and their receptors.

## Competing interests

The author(s) declare that they have no competing interests.

## Authors' contributions

MJB was responsible for the conception of the project, acquisition and analysis of the data, interpretation of results and writing of the manuscript. DB, BB and GS were responsible for the conception and design of the project, intellectual input, interpretation of results, and helped to draft the manuscript. RH was responsible for the clinical aspects of this project, including the assessment of MS patients. All authors read and approved the final manuscript.

## Pre-publication history

The pre-publication history for this paper can be accessed here:



## Supplementary Material

Additional File 1 Primers for the amplification of CC chemokine genes. This table presents the list of all primers used for pooled DNA sequencing. Click here for file

Additional File 3 Conditions for seven SNPs genotyped by SNaPshot. This table describes the oligonucleotides used for the SNaPshot genotyping, including reaction conditions. Click here for file

Additional File 2 Summary of SNPs identified and analysed by sequencing of CC chemokine genes in DNA pools. This table is an extended version of Table 1, which describes the minor allele frequency and estimated relative risk data for all SNPs identified by pooled DNA sequencing. Click here for file
