# Supplementary material for: An investigation of polymorphisms in the 17q11.2-12 CC chemokine gene cluster for association with multiple sclerosis in Australians
Source: BMC Med Genet. 2006 Jul 26;7:64. doi: 10.1186/1471-2350-7-64 (PMC1550395; doi:10.1186/1471-2350-7-64)
Supplement: Additional File 1 — Primers for the amplification of CC chemokine genes. This table presents the list of all primers used for pooled DNA sequencing. [file 1471-2350-7-64-S1.doc]

**Supplementary Table 1** Primers for the amplification of CC chemokine genes

| **Primer** | **Sequence** | **PCR Product Length (bp)** |
| --- | --- | --- |
| **(a) Main primers** |  |  |
| CCL2 |  |  |
| PromoterA_FP | CCGAGATGTTCCCAGCACAG |  |
| PromoterA_RP | CTGCTTTGCTTGTGCCTCTT | 888 |
| PromoterA_SP | AGTATCTGGAATGCAGGCTC |  |
| PromoterB_FP | GTGCTTCTGTAGTTTCTGAGG |  |
| PromoterB_RP | TGCAGCATCCTGTGGATTTC | 987 |
| PromoterB_SP1 | CTCACAGAGTGCTTACTCTG |  |
| PromoterB_SP2 | GTTCTGCTAGGCTTCTATGA |  |
| Exon1_FP | GAAATCCACAGGATGCTGCA |  |
| Exon1_RP | CTGCTTGGATGGTCCATGAT | 311 |
| Exon1_SP | CCCACTTATCACTCATGGAAG |  |
| Exon2_FP | CTGGTGCTGATCATCTGGAT |  |
| Exon2_RP | TGCACTCTCTGACTCTAGGT | 474 |
| Exon2_SP | CAGCATAGGAAACTGAGTCA |  |
| Exon3_FP | TTGAAGTCAGGATGGCTCCA |  |
| Exon3_RP | CTTTGTCACCACCAAGCTCA | 685 |
| Exon3_SP | GAATGGGCTGCACTTCTAGA |  |
| CCL7 |  |  |
| Promoter_FP | TGTTCCCTAACCTTGCACCT |  |
| Promoter_RP | GGGAGGATAGGAAATGACAG | 690 |
| Promoter_SP | GTAGAGGAAGACCACAGTATG |  |
| Exon1_FP | CTGTCATTTCCTATCCTCCC |  |
| Exon1_RP | GAAGCCTCCTGTCTTTGATC | 547 |
| Exon1_SP | AGTGCACTGGCTCAGCAGAT |  |
| Exon2_FP | GATCAAAGACAGGAGGCTTC |  |
| Exon2_RP | AACCTAGGACTGAGGTGTGA | 617 |
| Exon2_SP1 | CGGAAGAGGAATTGATGGCT |  |
| Exon2_SP2 | TGAAATGCCTCAAAGGGCAG |  |
| Exon3_FP | TTCCTTCATCCCTGAGGCAT |  |
| Exon3_RP | AGCAGATGTCAGATCTGGAG | 871 |
| Exon3_SP | GCTAGACAGATTTCCCATAG |  |
| CCL11 |  |  |
| Promoter_FP | ACGAGAGGCTGCCTATTCTA |  |
| Promoter_RP | CTGACCAGATTCCAGATCCT | 609 |
| Promoter_SP | AGTGCCTCAGAAGTCAAGAT |  |
| Exon1_FP | AGGATCTGGAATCTGGTCAG |  |
| Exon1_RP | AAGCTGTCCTGGAGTCGAAT | 596 |
| Exon1_SP | CACACTGTCTGCTCCCTATA |  |
| Exon2_FP | ACAGCATCCTCCAGACACAT |  |
| Exon2_RP | GAGACTCTTGGGTTAGTTGC | 496 |
| Exon2_SP | TTCAGAGCAGAGGGATTCTC |  |
| Exon3_FP | GCAACTAACCCAAGAGTCTC |  |
| Exon3_RP | GGAAAGGAATGGTGGCAAAG | 904 |
| Exon3_SP | AGAGTCTCATCCTTCCTCCT |  |
| CCL8 |  |  |
| Promoter_FP | GATCAGTGGTAGCCAGAGAT |  |
| Promoter_RP | CTTTTGCTTAGGCTCTGTGG | 672 |
| Promoter_SP | GGATTGTGCTCAGCAAAGTG |  |
| Exon1_FP | CCACAGAGCCTAAGCAAAAG |  |
| Exon1_RP | TCCTCTTTACACAGCCACTG | 619 |
| Exon1_SP | ATTATCCCAGGATCTGGTGC |  |
| Exon2_FP | AACCCAGTCAACAATGACGG |  |
| Exon2_RP | CTCTTGCTGTCCAGGTGAAA | 522 |
| Exon2_SP | GTGACTTCTTGGTCTTCTCC |  |
| Exon3_FP | AATATCTGTAGCCAGGACCC |  |
| Exon3_RP | TACAGGAGCACTGATTGCCA | 666 |
| Exon3_SP | CTTCCTTCTGGAGCTTCTTC |  |
| CCL13 |  |  |
| Promoter_FP | TAGTGGAATGCAGAGGGATG |  |
| Promoter_RP | GAAAGAGTGCTGTGCTAAGG | 591 |
| Promoter_SP | GGGATGAGTATTCCAGGAAG |  |
| Exon1_FP | CCTTAGCACAGCACTCTTTC |  |
| Exon1_RP | GCCTGGGCAACATAATGAGA | 569 |
| Exon1_SP | AGCTCCTTTATGGCTTCCCA |  |
| Exon2_FP | AAACTCTGCAGATCTGGGAC |  |
| Exon2_RP | CATCACACAGTCTGGCTAGA | 519 |
| Exon2_SP | CTGTGACATGTCTCCACCAT |  |
| Exon3_FP | TAGCAGATGCCAGGATCAGT |  |
| Exon3_RP | TGTACATCTGCCTTCCACTG | 848 |
| Exon3_SP | TGTCCACACACCTCCTACTT |  |
| CCL1 |  |  |
| Promoter_FP | TGAGTTTGCACCATGGCACT |  |
| Promoter_RP | ATAGGGACAGGGAACAGTTG | 857 |
| Promoter_SP | CAGCGACCATGTGGTAAACT |  |
| Exon1_FP | AGTTTACCACATGGTCGCTG |  |
| Exon1_RP | GGTCTTGCCTTCTGACTCTA | 582 |
| Exon1_SP | CAACTGTTCCCTGTCCCTAT |  |
| Exon2_FP | TTACCTAGCCCCAGAACAAG |  |
| Exon2_RP | AGTTACCTGTCCACGGTCAC | 633 |
| Exon2_SP | TGGCTGATGAGGCAGAGCTAT |  |
| Exon3_FP | CAGCGTGCAAACCACACATC |  |
| Exon3_RP | AAAGGCAGTCACTGAGAGCT | 567 |
| Exon3_SP | ATTTACAGAGGGGAGCCCAA |  |
| CCL5 |  |  |
| PromoterExon1_FP | ACAGTGTGAGTGTGCTCACCT |  |
| PromoterExon1_RP | CAAGGTGTGGGACAGTCATT | 782 |
| PromoterExon1_SP1 | TATATCCAGAGGACCCTCCT |  |
| PromoterExon1_SP2 | TAGGCATTCTAGGCAGAGTC |  |
| Exon2_FP | AGATGAGGCATTGGAATCCC |  |
| Exon2_RP | TTGGAGTCCTGGGAACATAG | 475 |
| Exon2_SP | CCAGCACAATGTCAAGTGTG |  |
| Exon3_FP | CATGCATTTGTGTGAAGGCG |  |
| Exon3_RP | TGGGGTTTCATCATGTTGGC | 558 |
| Exon3_SP | CCTTAATCCACTTCATCCCC |  |
| CCL16 |  |  |
| Promoter_FP | TTGTCAGGTGCACAGAGTTG |  |
| Promoter_RP | TGGTGCAGAGCTGAATTCAG | 607 |
| Promoter_SP | ATCAGCGAGCTGGCTACACCTA |  |
| Exon1_FP | CTGAATTCAGCTCTGCACCA |  |
| Exon1_RP | TCATTCCTGCTCACCACGCTT | 491 |
| Exon1_SP | TTACCCCAGAAAGATCCGGA |  |
| Exon2_FP | GGGTAGTGACTGTATCTTCC |  |
| Exon2_RP | CTGCTTCTAGCATGGAGACCT | 548 |
| Exon2_SP | TGGAGACCTCTTGATCCCAT |  |
| Exon3_FP | CATAAGGTGAGCTCCATGTG |  |
| Exon3_RP | AGATCATCTGGAAGGCTGGA | 877 |
| Exon3_SP1 | AAAGTTAAGGGACGTGGGCA |  |
| Exon3_SP2 | AAGTGAGCCAAGATCACACC |  |
| CCL14 |  |  |
| Promoter_FP | GCTTTTACAGAGCTCCGTTG |  |
| Promoter_RP | AGGCATGAACATGAGCACAG | 515 |
| Promoter_SP | GAGAACACAGACTTGGAAGC |  |
| Exon1_FP | CTGTGCTCATGTTCATGCCT |  |
| Exon1_RP | TCTCATCTCCATCCTGCTCA | 559 |
| Exon1_SP | GAGGCATCACTGCTTTCCTA |  |
| Exon2_FP | TTAGGAGCAGTGTCTTGAGC |  |
| Exon2_RP | ATCACAATGGCCTCAGAGGT | 476 |
| Exon2_SP | CAGTGAGTGCAGCATTGCTT |  |
| Exon34_FP | AGCTTGCAGGAGCAGCCAAT |  |
| Exon34_RP | TCAGTGCATGGCCAATGAGT | 877 |
| Exon34_SP1 | CATACTCTTCGGTTCCTAGG |  |
| Exon34_SP2 | AGAAGAAGGCAAGAGGGCACTT |  |
| CCL15 |  |  |
| Promoter_FP | TGGTTAGAACTTGACGTGGC |  |
| Promoter_RP | TGGAGAGATTCAAGTGCCTG | 799 |
| Promoter_SP | GGCCAGCTTGACTCATTCTT |  |
| Exon1_FP | CAGGCACTTGAATCTCTCCA |  |
| Exon1_RP | GGAACAAGTGAGACAGTGATC | 1093 |
| Exon1_SP1 | GGTCTCTCACTCTGCCTTAT |  |
| Exon1_SP2 | CAGACAGAGCCAGGTCTATA |  |
| Exon2_FP | CCGTGTTAGGTTCAGTTGCT |  |
| Exon2_RP | CCCAGGGTTTTGCCATGTGT | 614 |
| Exon2_SP | GGTCTCTGAAAAGTCTCAGG |  |
| Exon34_FP | GAGGGTCTGAAGCAGGGAGA |  |
| Exon34_RP | GCTCAGCAGTTGCTTCAGTT | 921 |
| Exon34_SP1 | AGCCTGTGGGACTCCTTAAT |  |
| Exon34_SP2 | AAGCAAGCACAAGGCTTCCCA |  |
| CCL23 |  |  |
| Promoter_FP | ATTGACCTGGCTGTTCCTGA |  |
| Promoter_RP | GACTTCCCGAGTTGTTCCAA | 799 |
| Promoter_SP | GTGGTCTATGCTAACACCAG |  |
| Exon1_FP | TTGGAACAACTCGGGAAGTC |  |
| Exon1_RP | AACAGTGCCTGCCCTGTACATA | 785 |
| Exon1_SP | TCCACAAGGACAGTCCAAGT |  |
| Exon2_FP | CAGAGACACGACACAGATAG |  |
| Exon2_RP | ATGGATCCCATAGTGCAGAC | 853 |
| Exon2_SP | ACACAGCAACCTCCTGTAAC |  |
| Exon34_FP | CACAGCTCAACTCTGATGAG |  |
| Exon34_RP | CAGGAGATTAGGCTGCAGTGA | 993 |
| Exon34_SP1 | AGAAAGCAGGCACAAGTCTG |  |
| Exon34_SP2 | AGCTCAGGGCCATTGTGCTCTT |  |
| CCL18 |  |  |
| Promoter_FP | TCTTGCCTGATTGCCTTCAC |  |
| Promoter_RP | AACATTGGAGGTGCTGATGC | 809 |
| Promoter_SP | ATCATCTCTTCCTGGGCCTT |  |
| Exon1_FP | AGTAACCATAGGCAACCCTG |  |
| Exon1_RP | GGTTGAACTCTAGGAGACTG | 560 |
| Exon1_SP | GCATCAGCACCTCCAATGTT |  |
| Exon2_FP | CCTTCTGGATCTCTTTGTCC |  |
| Exon2_RP | TTAACTTCGGGGTTCACAGG | 486 |
| Exon2_SP | TCTCCCAGTTCTTCCTGACT |  |
| Exon3_FP | CCTGTGAACCCCGAAGTTAA |  |
| Exon3_RP | GACTGTAGAAGTACTTGGTCC | 750 |
| Exon3_SP | TGAGATGCCTGGGACAGAGAA |  |
| **(b) Additional primers** | | |
| CCL11 Exon3_FP2 | CACAAGTGAGTGTTCACTCC |  |
| CCL11 Exon3_RP2 | CTCAGTGTGGATTTCCCACA | 791 |
| CCL11 Exon3_RP3 | TCTTGGCCACAGCCATCTTT | 822 |
| CCL23_Exon4_SP | CCAAGATGTACCAGTGCACT |  |

FP – forward primer

RP – reverse primer

SP – sequencing primer
