# Supplementary material for: An investigation of polymorphisms in the 17q11.2-12 CC chemokine gene cluster for association with multiple sclerosis in Australians
Source: BMC Med Genet. 2006 Jul 26;7:64. doi: 10.1186/1471-2350-7-64 (PMC1550395; doi:10.1186/1471-2350-7-64)
Supplement: Additional File 3 — Conditions for seven SNPs genotyped by SNaPshot. This table describes the oligonucleotides used for the SNaPshot genotyping, including reaction conditions. [file 1471-2350-7-64-S3.doc]

**Supplementary Table 3** Conditions for seven SNPs genotyped by SNaPshot

| Locus | Primer sequence | Primer length (bp) | Amount of primer used for SNaPshot (pmol) |
| --- | --- | --- | --- |
| *CCL2* -2581A>G | TTTTGAAGTGGGAGGCAGACAGCT | 24 | 0.3 |
| *CCL15* 136+88C>T | CCTCAGGACCCTCTCATTCTCCTC | 24 | 0.2 |
| *CCL5* -471C>T | TTTTTTTTCCATGGATGAGGGAAAGGAG | 28 | 0.2 |
| *CCL11* 67G>A | TTTTTTTTTTTTGGCTTACCTGGCCCAG | 28 | 0.4 |
| *CCL23* -289A>C | TTTTTTTTTTTTATGAATCTCCCAGAGGGAAG | 32 | 0.2 |
| *CCL15* -1284A>C | TTTTTTTTTTTTTTTCAGGTTGGTCTCGAACT | 32 | 0.4 |
| *CCL2* -2138A>T | TTTTTTTTTTTTTTCAGGGGAAACCTCTCTCTAATC | 36 | 0.3 |
