# Supplementary material for: An investigation of polymorphisms in the 17q11.2-12 CC chemokine gene cluster for association with multiple sclerosis in Australians
Source: BMC Med Genet. 2006 Jul 26;7:64. doi: 10.1186/1471-2350-7-64 (PMC1550395; doi:10.1186/1471-2350-7-64)
Supplement: Additional File 2 — Summary of SNPs identified and analysed by sequencing of CC chemokine genes in DNA pools. This table is an extended version of Table 1, which describes the minor allele frequency and estimated relative risk data for all SNPs identified by pooled DNA sequencing. [file 1471-2350-7-64-S2.doc]

**Supplementary Table 2** Summary of SNPs identified and analysed by sequencing of CC chemokine genes in DNA pools

|  |  | **Estimated minor allele frequency** | **Estimated relative risk**  **(for minor allele frequency >0.15)** | | |
| --- | --- | --- | --- | --- | --- |
| Locus | **rsID** | **Control pool** | **HLA-DRB1*1501 positive pool** | **HLA-DRB1*1501 negative pool** | **Familial pool** |
| CCL2 |  |  |  |  |  |
| **-2581A>G** | **1024611** | **0.5** | **0.9** | **0.8** | **0.7** |
| -2411G>C | 3917903 | <0.1* | Undetectable | Undetectable | Undetectable |
| **-2138A>T** | **1024610** | **0.3** | **1.2** | **1.5** | **1.5** |
| -928G>C | 3760396 | 0.1 | - | - | - |
| -362G>C | 2857656 | 0.5 | 1.0 | 1.0 | 0.8 |
| 77-108C>G | 2857657 | 0.2 | 1.0 | 1.3 | 1.2 |
| 105 T>C (Silent)^+^ | 4586 | 0.4 | 0.9 | 0.8 | 0.7 |
| *65C>T | 13900 | 0.2 | 0.9 | 1.0 | 0.8 |
| CCL7 |  |  |  |  |  |
| 107-362C>T | 3091237 | <0.1 | - | - | - |
| 107-293C>T | 3091321 | 0.2 | 1.2 | 0.5 | 1.3 |
| CCL11 |  |  |  |  |  |
| -576T>C | 4795896 | 0.1 | - | - | - |
| **-488C>A** | **17735961** | **0.2** | **1.2** | **1.2** | **1.3** |
| -384A>G | 17809012 | 0.4 | 1.1 | 0.9 | 1.2 |
| **67G>A (A>T)^+^** | **3744508** | **0.1** | **-** | **-** | **-** |
| 76+229A>T | 1860184 | 0.3 | 0.9 | 1.0 | 0.8 |
| *411A>G | 1019109 | 0.1 | - | - | - |
| CCL8 |  |  |  |  |  |
| **-572C>T** | **3138035** | **0.3** | **1.1** | **1.0** | **1.2** |
| -390G>A | Novel | <0.1 | - | - | Undetectable |
| 194+139A>G | 3138036 | 0.2 | 0.9 | 0.7 | 0.9 |
| 195-77T>C | 3138037 | 0.1 | - | - | - |
| ^†^205A>C (K>Q)^+^ | 3138038 | 0.2 | 0.8 | 0.8 | 0.7 |
| CCL13 |  |  |  |  |  |
| -798C>T | 3136675 | Undetectable | - | - | - |
| -257A>G | 3136676 | <0.1 | - | Undetectable | Undetectable |
| 77-105T>C | 159313 | 0.3 | 1.2 | 1.0 | 1.2 |
| 192-54A>G | 2072069 | 0.4* | 1.0* | 0.8* | 1.0* |
| CCL1 (-) |  |  |  |  |  |
| -778G>A | 159274 | <0.1 | Undetectable | Undetectable | Undetectable |
| 76+272T>G | 544325 | 0.4* | 1.2* | 0.9* | 1.1* |
| 188+298A>C | 3138031 | 0.2 | 0.8 | 0.8 | 0.9 |
| *86C>T | 3136682 | <0.1 | Undetectable | Undetectable | Undetectable |
| CCL5 (-) |  |  |  |  |  |
| **-471C>T** | **2107538** | **0.2** | **1.2** | **1.3** | **1.1** |
| CCL16 (-) |  |  |  |  |  |
| **-595C>A** | **854680** | **0.1** | **-** | **-** | **-** |
| 77-226A>G | 917015 | 0.3 | 1.2 | 0.9 | 1.2 |
| *355G>A | 2516845 | 0.1 | - | - | - |
| CCL14 (-) |  |  |  |  |  |
| **-649T>A** | **854682** | **0.2*** | **1.1*** | **1.0*** | **1.4*** |
| -322G>C | 854683 | 0.2* | 1.4* | 1.1* | 1.5* |
| CCL15 (-) |  |  |  |  |  |
| **-1284A>C** | **854628** | **<0.1** | **-** | **Undetectable** | **-** |
| -1019C>T | 854627 | 0.2* | 1.0* | 0.7* | 1.1* |
| -997A>C | 854626 | <0.1 | - | - | - |
| -11G>A | 2293788 | <0.1* | - | - | - |
| 71G>A (T>I)^+^ | 854625 | <0.1 | Undetectable | Undetectable | Undetectable |
| **136+88C>T** | **Novel** | **0.3** | **0.8** | **0.6** | **0.5** |
| 136+132T>C | 864104 | 0.2 | 0.9 | 1.2 | 0.8 |
| 137-105T>C | 2075746 | 0.5 | 0.9 | 0.8 | 0.9 |
| CCL23 (-) |  |  |  |  |  |
| -727A>C | 854656 | 0.2* | 0.9* | 0.7* | 1.1* |
| **-289A>C** | **854655** | **0.2*** | **0.7*** | **0.5*** | **1.0*** |
| **316T>C (M>V)^+^** | **1003645** | **0.2*** | **1.0*** | **0.7*** | **1.0*** |
| CCL18 |  |  |  |  |  |
| -429A>T | 1967293 | <0.1 | - | - | - |
| -86A>G | 2015086 | 0.1 | - | - | - |
| 67+14C>T | 2015070 | <0.1 | - | - | - |
| *94C>T | 14304 | 0.3* | 0.9* | 1.0* | 1.1* |

All SNPs classified relative to translation start site. Relative risks determined relative to control pool. SNPs in bold were individually genotyped in second stage

(-) Gene encoded in anti-sense direction; SNPs reclassified to account for this

^+^ Coding change in brackets

^†^ The CCL8 205 SNP was originally selected for genotyping by SNPLEX™, but failed the SNPLEX™ algorithm, and was replaced with the CCL8 -572 SNP

* Heterozygous individual correction applied

rsID: SNP identification number
